# The Bristol Scheme for Dealing with an Outbreak of Typhus

**Published:** 1942

**Authors:** R. J. Irving Bell

**Affiliations:** Senior Assistant Medical Officer of Health, Bristol


### THE BRISTOL SCHEME FOR DEALING WITH AN OUTBREAK OF TYPHUS.

Dr. R. J. Irving Bell, M.R.C.S., L.R.C.P., D.P.H.
*Senior Assistant Medical Officer of Health, Bristol.*

---

In Bristol we have two teams available to deal with cases of typhus, each consisting of the following :—

1 Medical Officer.

6 Nurses (2 of whom are reserves).

3 Sanitary Inspectors.

2 Drivers for ambulance and van.

2 Men to form disinfecting party.

In addition there is one disinfestation team, consisting of 8 men, stationed at the Disinfecting Depot, Feeder Road.

Those who have had some experience of fighting a typhus epidemic have found that the medical staff run a fair risk of infection—particularly when handling patients during admission to hospital—and so all the members of the teams have received :—

(*a*) Protective inoculation (although at present the immunity given by the vaccine is doubtful).

(*b*) Protective clothing (described later). Also

(*c*) The hair of the head will be cut as short as possible and shaved if necessary on other parts of the body.

The control of the spread of typhus is bound up entirely with the destruction of lice and their eggs, and for this purpose we have an efficient insecticide—Lethane 384 (Special). Lethane in petroleum oil is sprayed on the protective clothing, inside and outside, before and after contact with infected patients.

Our first warning of typhus may come from :—

i. A general practitioner who reports a doubtful case.

ii. Dr. Cooper at Canynge Hall, who may discover a positive Weil-Felix reaction.

iii. From Bristol Airport.

iv. From a ship arriving at Avonmouth or Bristol Docks.

The patient will be visited by one of the team of medical officers with a consultant if one is available. If the diagnosis of typhus is confirmed or the case is labelled " suspected " then the team with the mobile disinfecting unit proceeds to the house. One or two nurses go with the patient to the small-pox block at Ham Green Hospital and the patient is admitted there after being disinfested in a special section provided. The nurses who accompany the patient cleanse themselves and change clothes, then remain at the Hospital for further orders. The driver of the ambulance and one Sanitary Inspector (acting as attendant) are also disinfected.

The other nurses in the team will be examining female contacts (if any) at the house of the patient, and the Sanitary Inspectors will prepare the house for disinfection by the disinfecting team and collect bedding, etc., for stoving or destruction. Contacts will be removed and quarantined. One of the Sanitary Inspectors will find out where members of the patient's family work and where contacts reside and transmit this information to the Health Department for field investigation which will be carried out by team 2. This team will do all field investigation other than at the infected premises whilst team 1 will do all work at the infected premises, and between them and the Hospital. As need arises each team may be augmented and the teams can change places for relief purposes. After the premises have been cleansed and disinfested and contacts removed and quarantined, all members of the team will themselves be cleansed and disinfested. This plan of action will be similarly adopted when dealing with infected aircraft or ships.

The protective clothing worn by the teams is a one-piece garment of unbleached calico, the legs being continued into the feet as in fishing waders. It is completely closed except at the back (where it is zip-fastened), round the face and at the wrists. Gum-boots and rubber gloves are worn also.

This is an outline of the steps which will be taken if and when we may have to deal with typhus in Bristol. There are many other details connected with this work and these have been worked out during practice of the teams.

